# Heterosis for capsacinoids accumulation in chili pepper hybrids is dependent on parent-of-origin effect

**DOI:** 10.1038/s41598-022-18711-w

**Published:** 2022-08-24

**Authors:** Emmanuel Rezende Naves, Federico Scossa, Wagner L. Araújo, Adriano Nunes-Nesi, Alisdair R. Fernie, Agustin Zsögön

**Affiliations:** 1 Departamento de Biologia Vegetal, Universidade Federal de Viçosa, Viçosa, MG CEP 36570-900 Brazil; 2 Max-Planck-Institute for Molecular Plant Physiology, 14476 Potsdam, Germany; 3 Council for Agricultural Research and Economics (CREA), Research Centre for Genomics and Bioinformatics (CREA-GB), 00178 Rome, Italy

**Keywords:** Plant sciences, Secondary metabolism

## Abstract

Heterosis for agronomic traits is a widespread phenomenon that underpins hybrid crop breeding. However, heterosis at the level of cellular metabolites has not yet been fully explored. Some metabolites are highly sought after, like capsaicinoids found in peppers of the *Capsicum* genus, which confer the characteristic pungent (‘hot’) flavour of the fruits. We analysed the metabolic profile of the fruit placenta and pericarp of inter- and intra-specific hybrids of two species of *Capsicum* peppers, *C. chinense* (cv. Habanero and cv. Biquinho) and *C. annuum* var. *annuum* (cv. Jalapeño and cv. Cascadura Ikeda) in complete diallel crosses with reciprocals. The parents and hybrids were grown in a glasshouse and the profile of primary metabolites (sugars, amino acids and organic acids) and capsaicinoids was generated via gas chromatography–time of flight-mass spectrometry (GC–TOF-MS) and ultra-performance liquid chromatography coupled to a mass spectrometer (UPLC-MS), respectively. We found considerable heterotic effects specifically for capsaicinoids accumulation in the fruit placenta of the hybrids, including those derived from non-pungent parents. Furthermore, a large fraction of fruit primary metabolism was influenced by the specific cross combination, with marked parent-of-origin effects, i.e. whether a specific genotype was used as the pistillate or pollen parent. The differences in metabolite levels between the hybrids and their parents provide a snapshot of heterosis for primary and secondary metabolites and may contribute to explain the manifestation of whole-plant heterotic phenotypes.

## Introduction

*Capsicum* is a genus of the nightshade family (Solanaceae) comprising more than 35 species^1^. Five species (*C. annuum* var. *annuum*, *C. chinense*, *C. baccatum*, *C. frutescens* and *C. pubescens*) were independently domesticated in Central and South America and are cultivated today across subtropical and temperate climates all over the world^2^. Pepper fruits (‘pods’) show a large diversity of shape, color and taste, so that different market types exist, bred specifically for fresh consumption (sweet peppers), fresh processing (e.g. sauce, paste), dried spice^3^, oleoresin extraction^4^ or ornamental purposes^5^. The consumers’ preferences for diverse organoleptic properties have also produced a wide variation of pungengy (‘heat’) levels, ranging from prohibitively hot fruits to mild or totally sweet forms^6^. Pungency is conferred by capsaicinoids, a class of vanillylamides, which accumulate in variable amounts during ripening in the fruit placenta of hot varieties^7^. Capsaicinoids, capsinoids^8^ and other pepper secondary metabolites have a variety of uses in the agrifood, cosmetic and pharmaceutical industries as replacements for synthetic additives^9^.

Peppers are naturally allogamous (i.e. outcrossing) species^10^, however, commercial pepper varieties are managed and bred as fully autogamous^11^. Hybridization between pepper varieties or species was generally used for fundamental research to identify genes of interest, but more recently it has become commonplace as a breeding tool per se^12^. Hybridization breeding allows the combination of dominantly inherited traits, including disease resistance and agronomic traits^13^. Another advantage of hybrids is that they can display considerable hybrid vigour, or heterosis^14^. Heterosis is a complex phenomenon, which has been fundamental in improving the yield of many annual crops, such as maize (*Zea mays*), sugar beet (*Beta vulgaris*), rapeseed (*Brassica napus* var. *napus*), rye (*Secale cereale*), rice (*Oryza* spp) and cotton (*Gossypium* spp), but whose underlying molecular mechanisms have defied explanation^15^.

While generally less investigated, the analysis of metabolic variation may also point to important differences involved in the manifestation of heterotic phenotypes^16^. Metabolic models of heterosis predict that an optimal concentration of enzymes and metabolites is reached in hybrids, in contrast to what may happen in inbreds lines^17^. In any case, the study of metabolite variation in hybrids may be of interest not only to provide mechanistic insights on heterosis but also to inform breeding for higher content of metabolites of interest. Capsaicinoids are highly valuable secondary compounds but notoriously unreliable in their concentration, which is strongly influenced by the environment^18^. Novel heterologous platforms for capsaicinoid production have been proposed^7,19^, but achieving a high and consistent level of pungency in hot peppers pods represents a more immediate and convenient avenue for crop breeders.

The aim of this work was to analyse primary metabolite (sugars, amino acids and organic acids) and capsaicinoid accumulation patterns in hybrids between the highly pungent (hot) *C. chinense* cv. Habanero and *C. annuum* var. *annuum* cv. Jalapeño and non-pungent (sweet) commercial cultivars *C. chinense* cv. Biquinho and *C. annuum* var. *annuum* cv. Cascadura Ikeda. Understanding the metabolic consequences of hybridization would be desirable to inform the use of these varieties in hot pepper breeding programmes. We performed the metabolic profiling of fruit placentas and pericarps from full diallel (reciprocal) crosses between cultivars of the same or different species. The results point to a large influence of the specific cross combination on fruit metabolite levels in the hybrid progenies. In particular, most primary metabolites, in both pericarp and placenta, accumulate to different levels with respect to their parents, showing distinct patterns of non-additivity. Furthermore, capsacinoid accumulation shows a highly heterotic pattern in certain combinations, leading to transgressively higher heat levels compared to the parental lines. We discuss these results within the context of the exploitation of heterosis to increase the accumulation of valuable secondary metabolites in horticultural crops through hybridization.

## Results

**Figure 1:**
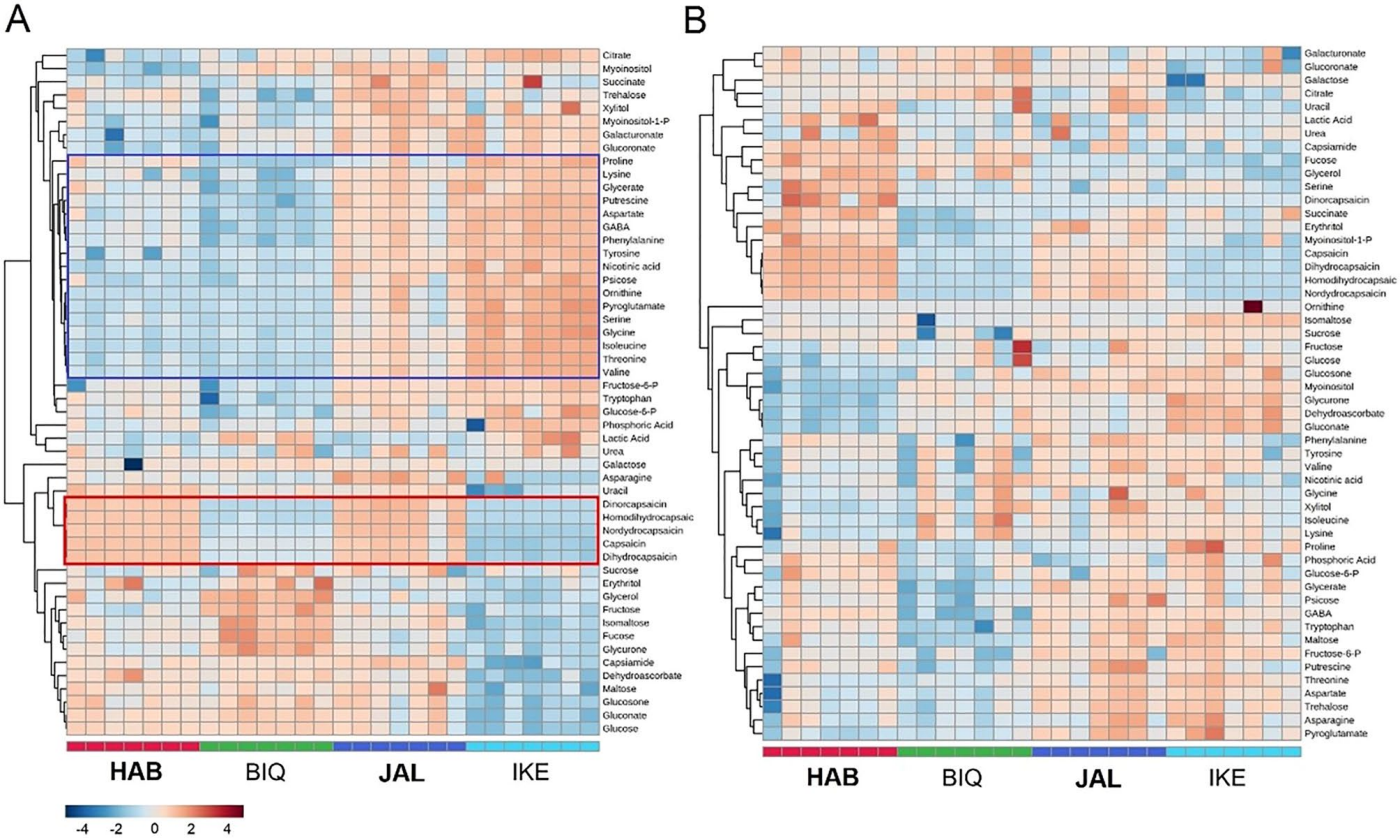
Hierarchical clustering and heat map of primary metabolites in (**A**) the fruit placenta (PLA) and (**B**) pericarp (PER) of *C. chinense* cv. Habanero (HAB), *C. chinense* cv. Biquinho (BIQ), *C. annuum* cv. Jalapeño (JAL) and *C. annuum* cv. Cascadura Ikeda (IKE). The pungent genotypes are shown in boldface. Each column represents a genotype, the seven subdivisions within each column represent individual plants (n = 7). Rows represent traits. The color key indicates the relative trait value normalized by glog transformation and autoscaled (mean-centered and divided by the standard deviation of each variable). In the placenta all capsaicinoids cluster together (highlighted in red) and most amino acids cluster together (highlighted in blue).

We carried out a detailed metabolic profiling of metabolites in parental genotypes and their hybrids in order to analyse the reciprocal heterotic effects on the entire pool of capsaicinoid precursors. As expected, the placental tissues of the pungent parents from both *C. chinense* (cv. Habanero, HAB) and C*. annuum* (cv. Jalapeño, JAL) showed high accumulation of the main capsaicinoids, in contrast to the sweet peppers (*C. chinense* cv. Biquinho, BIQ and *C. annuum* cv. Cascadura Ikeda, IKE) (Fig. 1). Capsaicinoids accumulation pattern was discernible and clear-cut between hot and sweet varieties, while amino acids clustered together and displayed a signature that was mostly associated with species rather than variety in the placenta (higher accumulation was observed in *C. annuum* varieties than in *C. chinense* ones) (Fig. 1A). The metabolic signature of the pericarp, on the other hand, was less consistent. Residual capsaicinoid levels were also found in the hot varieties, and metabolite accumulation displayed higher variation between individual replicates than for the placenta (Fig. 1B).

**Figure 2:**
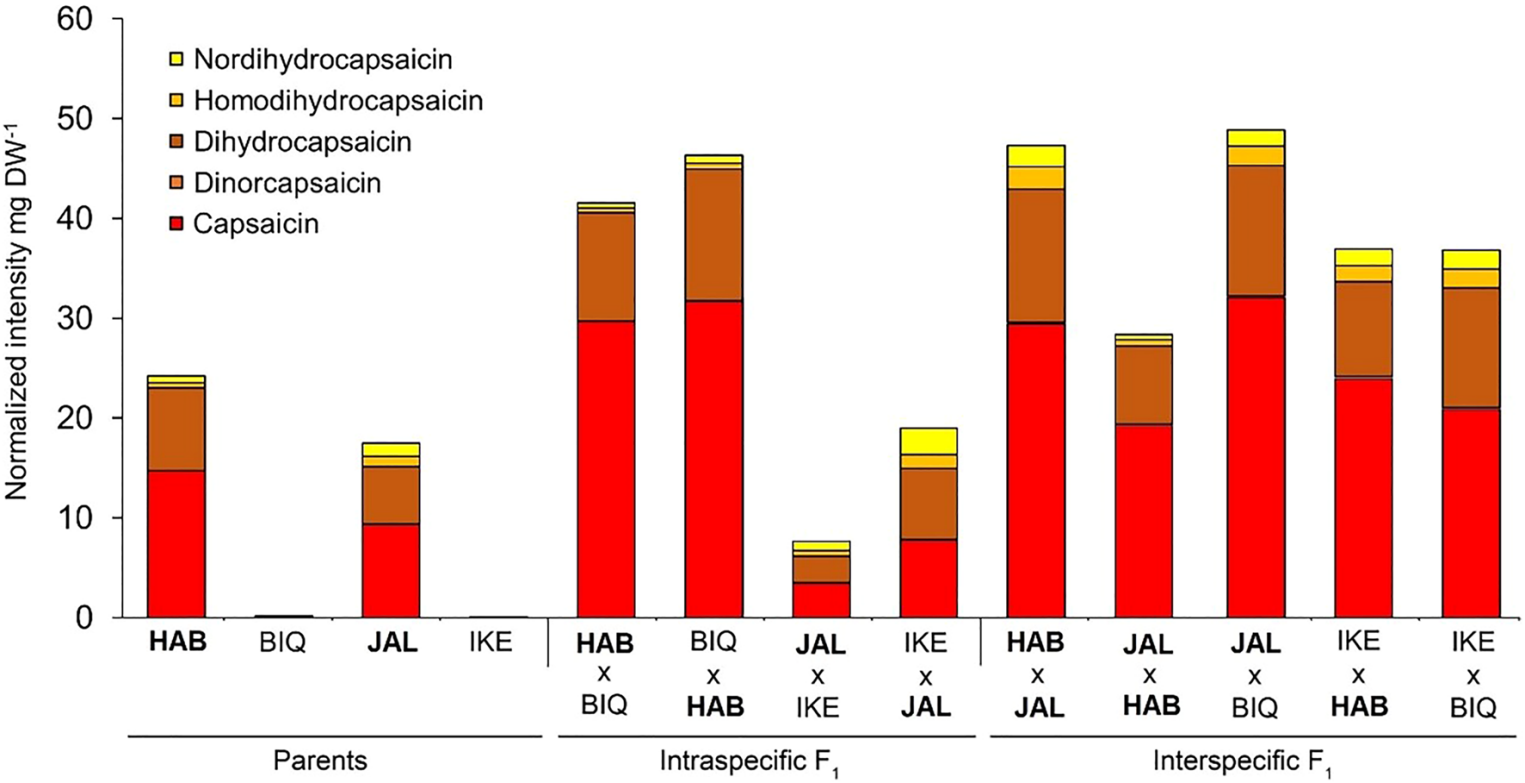
Relative capsaicinoid content in the placenta of intra and interspecific hybrids of *Capsicum*. Parents: *C. chinense* cv. Habanero (HAB), *C. chinense* cv. Biquinho (BIQ), *C. annuum* cv. Jalapeño (JAL) and *C. annuum* cv. Cascadura Ikeda (IKE). The pungent genotypes are shown in boldface. Each column represents a genotype (n = 7).

The most abundant capsaicinoids were capsaicin (C_17_H_27_NO_3_, [M + H]^+^ = 306.206356, dinorcapsaicin (C_16_H_23_NO_3_, [M + H]^+^ = 278.175056) and dihydrocapsaicin (C_18_H_29_NO_3_, [M + H]^+^ = 611.405446), which accounted for over 90% of total capsaicinoid content in all genotypes (Fig. 2). Small amounts of homodihydrocapsaicin and nordihydrocapsaicin were also detected. As expected, HAB had the higher total capsaicinoids level in the placenta, followed by JAL, whereas these compounds were not detected in the sweet peppers BIQ and IKE (Fig. 2). Notably, all hybrids were pungent, regardless of their parental genotypes and, except for the intraspecific hybrids of *C. annuum,* all combinations displayed significant degrees of both relative mid-parental heterosis (rMPH) and relative best-parent heterosis (rBPH) for all of the main capsaicinoids (Table 1).

**Table 1:** Relative mid-parental heterosis (rMPH) and best-parental heterosis (rBPH) for the accumulation of the three main capsaicinoids (capsaicin, dihydrocapsaicin and nordydrocapsacin) and for the total capsaicinoids in the placenta of fully ripe fruits in intraspecific and interspecific hybrids of *Capsicum*.

Genotype	Hybrids								
	Intraspecific F_1_				Interspecific F_1_				
	*HAB × *BIQ	BIQ × *HAB*	*JAL × *IKE	IKE × *JAL*	*HAB × JAL*	*JAL × HAB*	*JAL × *BIQ	IKE × *HAB*	IKE × BIQ
	Capsaicin								
rMPH	300.70**	328.46**	− 34.7	54.59	132.16**	168.11**	347.05**	184.16**	39,493.63**
rBPH	101.65**	115.63**	− 67.35**	− 22.69	99.90**	130.85**	125.54**	42.10**	20,027.80**
	Dihydrocapsaicin								
rMPH	161.43**	217.84**	− 31.96	127.22**	79.89**	75.87**	186.84**	189.16**	76,568.97**
rBPH	31.03**	59.30**	− 65.98**	13.62	61.71**	58.10**	43.85**	44.59*	39,402.03**
	Nordyhidrocapsaicin								
rMPH	58.92**	131.32**	− 8.59	307.87**	94.36**	47.29*	117.87**	453.92**	46,169.57**
rBPH	− 20.28**	16.04*	− 54.29**	103.94**	42.15**	7.73	9.1	176.96**	23,034.78**
	Total capsaicinoids								
rMPH	2745.10**	284.14**	− 31.64	100.57**	111.88**	129.49**	272.43**	193.87**	45,860.17**
rBPH	1322.81**	93.03**	− 65.82**	0.300	89.74**	105.52**	87.39**	46.95**	23,034.78**

Parents: *C. chinense* cv. Habanero (HAB), *C. chinense* cv. Biquinho (BIQ), *C. annuum* cv. Jalapeño (JAL) and *C. annuum* cv. Cascadura Ikeda (IKE). The pungent genotypes are shown in italicface.

We found considerable heterotic effects specifically for capsaicinoids accumulation in the placenta of both intra- and interspecific hybrids, including those derived from non-pungent parents (BIQ and IKE) (Table 1). The intraspecific cross of the hot (HAB) and sweet (BIQ) varieties of *C. chinese* led to roughly twice the concentration of capsaicinoids relative to the hot parent (Fig. 2), with little effect of parent of origin. In contrast, the hybrids between hot (JAL) and sweet (IKE) varieties of *C. annuum* did not produce significant increases in pungency relative to JAL when this genotype was used as pollen donor, but led to a reduction in half in capsaicinoids concentration in the reciprocal (JAL × IKE) cross. Heterotic effects in capsaicinoids accumulation in the placenta were more evident when analysing interspecific hybrids (Fig. 2). Heterosis in the HAB × JAL hybrids was strongly depedent on parent of origin: whereas using HAB as a pistilliate (i.e. female) parent led to doubling of the pungency of the hottest parent (HAB), the hybrids derived from the reciprocal cross showed similar capsaicinoids levels as HAB. Lastly, in the three combinations of crosses for which reciprocal hybrids were not viable (JAL × BIQ; IKE × HAB; IKE × BIQ), considerable heterosis for capsaicinoids accumulation was observed: the JAL × BIQ hybrid had triple the levels of the hot parent (JAL), the IKE × HAB hybrid around 50% more than HAB and, remarkably, the hybrid derived from non-pungent parents (IKE × BIQ) displayed high accumulation of capsaicinoids (Fig. 2).

By contrast, the pericarp metabolites of interspecific hybrids showed mid- to strong hybrid depression, except for several amino acids from the HAB × JAL combination (glycine, threonine, branched-chain and aromatic amino acids, lysine, aspartate, asparagine, GABA, pyroglutamic acid and GABA). Here, most primary metabolites, whether from placenta or pericarp, generally showed non-additive accumulation patterns (i.e., their levels being significantly lower or higher with respect to the best parent), with few cases of reciprocal effects detected for amino acids in the placenta of the intraspecific cross of *C. chinense.*

**Figure 3:**
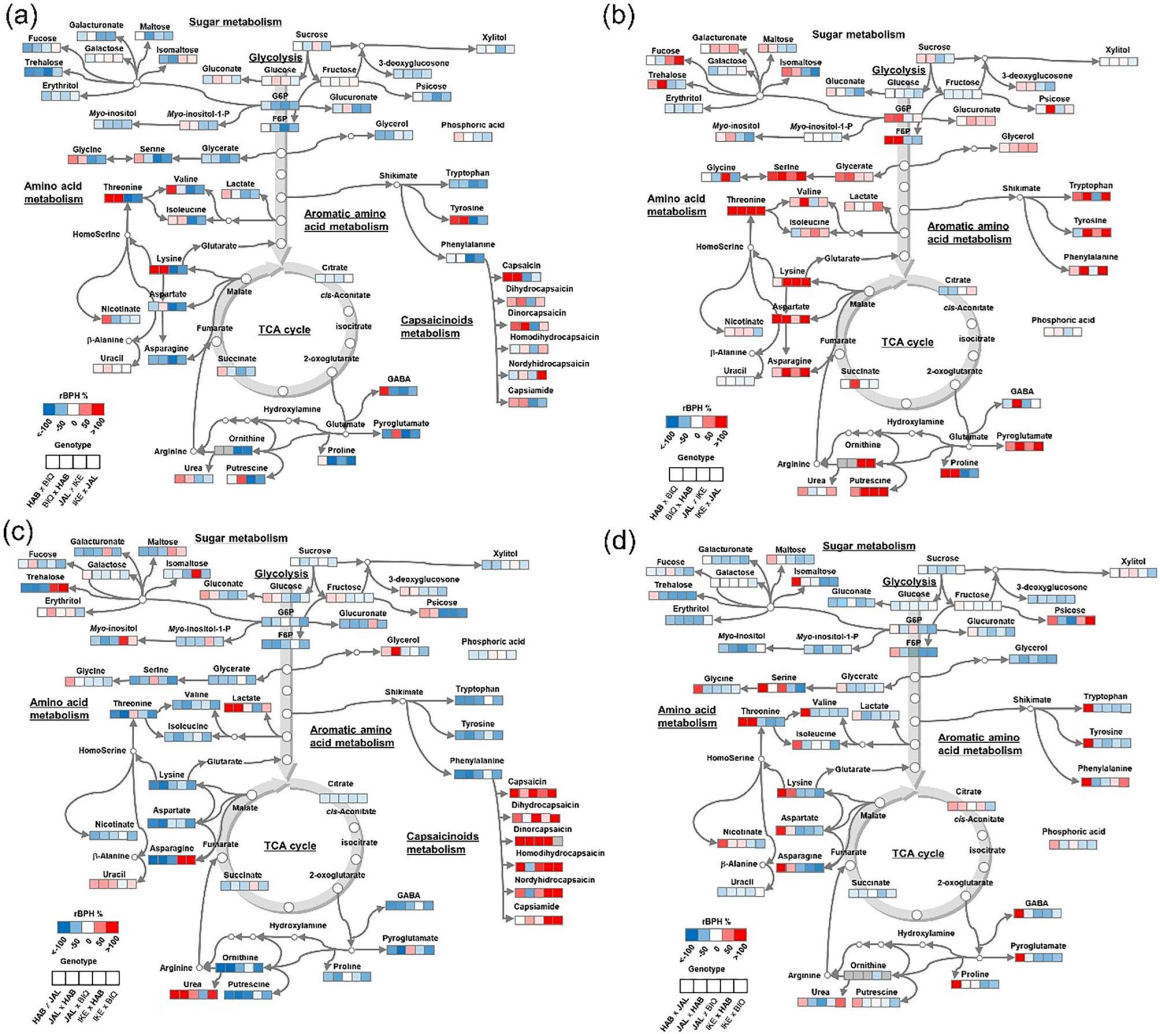
Heat map of primary metabolites and capsaicinoids in the fruit placenta (**a**) and pericarp (**b**) of intra-specific and (**c**) placenta and (**d**) pericarp or interspecific hybrids of *C. chinense* cv. Habanero (HAB), *C. chinense* cv. Biquinho (BIQ), *C. annuum* cv. Jalapeño (JAL) and *C. annuum* cv. Cascadura Ikeda (IKE). The pungent genotypes are shown in boldface. Each square represents a genotype (n = 7). The color key indicates the relative best parent heterosis (BPH) value (red = higher, blue = lower). The maps were generated using a custom-made template on MS-Powerpoint by color-coding the results obtained from Metaboanalyst 5.0 (https://www.metaboanalyst.ca/) and the heterosis calculations described in the “Materials and methods” section.

We then mapped the metabolite rBPH values onto the main pathways of primary metabolism and found that a few general trends emerged (Fig. 3). For the intraspecific crosses, where the diallel crosses allowed us to compare between the reciprocal effects of parents (i.e. differences in rBPH in hybrids derived from the same parents used in different male/female combinations), we observed marked parent-of-origin effects on the level of several metabolites. Among these, the most notable were amino acids, e.g., serine, valine, aspartate, GABA and pyroglutamic acid in the placenta of the fruits derived from the intraspecific *C. chinense* crosses (i.e., HAB × BIQ and its reciprocal). This reversion of the heterotic effect was not observed in the other intraspecific cross (the one derived from crossing JAL and IKE, both cultivars of *C. annuum*), where most of the primary metabolites, including amino acids, show the same sign of rBPH irrespective of whether JAL or IKE was used as the female parent (Fig. 3A,B).

Primary metabolites from the interspecific crosses (Fig. 3C,D) showed instead a more uniform pattern of hybrid depression (negative rBPH), except for trehalose and asparagine, which displayed considerable rBPH in the interspecific hybrids where IKE was used as female (Fig. 3C). In the pericarp (Fig. 3D), strong rBPH was detected for serine (along with its probable precursor, glycerate, given that serine biosynthesis in non-photosynthetic tissues mainly occurs through the phosphorylated pathway^20^, as well as for other amino acids derived from TCA intermediates (lysine, aspartate, asparagine and pyroglutamic acid). By contrast, the pericarp metabolites of interspecific hybrids showed mid- to strong hybrid depression, except for several amino acids from the HAB × JAL combination (glycine, threonine, branched-chain and aromatic amino acids, lysine, aspartate, asparagine, GABA, pyroglutamic acid and GABA). Thus, taken together, most primary metabolites in hybrids showed an accumulation pattern that was strongly dependent on tissue and parent of origin. On the other hand, capsaicinoids showed very consistent heterotic accumulation, except in the *C. annuum* intraspecific hybrids.

## Discussion

With the increasing challenge of unpredictable climate^21^, exploiting the full range of natural variation for peppers and other crops will be a suitable avenue to create novel, resilient varieties^22^. It can also pave the way for the creation of hybrids with altered combinations of visual and organoleptic traits, such as fruit size, shape, colour and flavour^23^. Exploitation of heterotic effects is highly desirable for hybrid breeding^24^. Here, we have shown that combinations of *C. annuum* var. *annuum* and *C. chinense* commercial varieties can produce valuable new metabolic phenotypes through heterosis in a manner that is strongly dependent on parent-of-origin. Further exploration of the genetic basis of these phenomena will contribute to the knowledge-based breeding of more resilient and appealing pepper varieties.

Hybrid breeding has been relatively underexploited in hot peppers, in large part due to a lack of knowledge about heterosis and parent-of-origin effects in different hybrid combinations^25^. *Capsicum* fruits are noted for their pungency (‘heat’), which is conferred by a class of metabolites collectively known as capsaicinoids, which accumulate specifically in the fruit placenta of hot pepper varieties and are highly sought by breeders^26^. Since capsaicinoids are synthesized from amino acidic precursors^27^, when analysing varieties with higher pungency it is also important to address associated changes in primary metabolism. Here, we found the expected pattern of capsaicinoids accumulation in the hot varieties used as parental genotypes for the hybrids: *C. chinense* cv. Habanero (HAB) had the highest ‘heat’ levels, followed by *C. annuum* cv. Jalapeño (JAL). Some capsaicinoids were detected in the fruit pericarp of these varieties. While generally accepted that the main site of capsaicinoids biosynthesis is the placental septum^28^, ‘super-hot’ varieties of peppers are known to elicit capsaicinoid biosynthesis in the fruit pericarp^29^. This is accompanied not only by anatomical changes and the formation of septum-like structures in the pericarp^29^ but also by associated transcriptional profiles leading to the activation of the capsaicinoids biosynthesis pathway^30–32^.

Although well-documented cases of heterotic phenotypes driven by a single gene have been reported, as in the case of the tomato *SINGLE FLOWER TRUSS* (*SFT*)^33^, heterosis is generally considered a phenomenon where large-scale changes in gene expression patterns are involved^34^. Large-scale transcriptome studies have detected varying degrees of non-additivity in gene expression (when the expression level in F_1_ hybrids ≠ midparent value), with cases of both repression (F_1_ < MPV) and activation (F_1_ > MPV) of genes with respect to the inbred parents. Non-additivity of gene expression has been proposed to underlie several yield heterotic phenotypes^14,35–37^ and has been generally found to be abundant, particularly in interspecific hybrids^15^. Recent work on *Brassica juncea* showed that primary and secondary metabolites display both additive and non-additive inheritance, depending on tissue type (buds vs. leaves) and developmental stage^38^. This behavior of non-additive heterotic activation in the F_1_ may be due to the complementation of non-functional alleles at distinct loci, with the restoration of a fully functional capsaicinoid pathway in the hybrids^39^.

A more detailed, systematic study on the metabolic effects of hybridization could therefore lead to the identification of parental genotypes for the production of superior hybrids with improved agronomic traits, although environmental factors may have a strong influence on the manifestation of the resulting phenotypes^40^. The potential heterosis of *Capsicum* hybrids has not been thoroughly investigated, although increased yield, biomass and pungency was found in some selected hybrids of *C. annuum*^41–43^. The existence of incompatibility barriers between species^44^ and the absence of a convenient male-sterility system to avoid self-fertilization^45^ have precluded large-scale implementation of commercial hybrids in hot peppers, which otherwise represent the fundamental breeding form in other crops^46^. However, with further advances in gene-editing technology^47^ and in the understanding of nucleus-cytoplasm interactions, novel high-throughput hybridisation systems may be realised to support hybrid breeding in the *Capsicum* genus.

## Conclusion

High capsaicinoid levels can be achieved in hybrid peppers via heterosis but with considerable parent-of-origin effect. Large-scale metabolic reprogramming is not observed, with only specific changes in some points of the capsaicinoid biosynthesis pathway intermediates. Further work should extend these observations to provide a framework for the predictive breeding of consistently hot hybrid peppers.

## Materials and methods

### Plant material and growth conditions

*Capsicum annuum* var. *annuum* (called *C. annuum* for brevity) cv. “Cascadura Ikeda” (IKE) and the pungent “Jalapeño” (JAL) were used for this study, while for *C. chinense*, the pungent cv. “Habanero” (HAB) and the sweet cv. “Biquinho” (BIQ) were grown. All seeds are commercially available (TopSeed, Agristar, São Paulo, Brazil). Intra- and interspecific F_1_ hybrids were created using a full diallel crossing scheme, by emasculating the flower buds before anthesis, and then transferring pollen from the selected male parent. The experiments were conducted in a greenhouse at the Federal University of Viçosa in Viçosa (642 m above sea level, 20° 45ʹ S, 42° 51ʹ W), MG, Brazil, between August 2018 and December 2019. The conditions in the greenhouse were: mean temperature 26/18 °C day/night, photoperiod 12 h/13 h winter/summer, peak midday irradiance 1200 µmol m^−2^ s^−1^, and daily irrigation to field capacity. Basic fertilization was done with 2 g L^−1^ NPK (10–10-10) and 4 g L^−1^ dolomitic limestone [CaMg(CO_3_)_2_]. Supplemental fertilization was provided fortnightly with leaf spray for minerals and micronutrients.

The varieties in this study were specifically selected for their range of divergent traits, including fruit size, shape and pungency: Habanero peppers are popular in the Yucatán peninsula of Mexico and some islands of the Caribbean and their fruits are characterized by strong pungency and fruity aroma. Cultivar Biquinho is a sweet pepper extremely popular in Brazil, where it is sold fresh or pickled as snack. Jalapeños are signature hot peppers which account for 30% of Mexico’s hot pepper production^48^, whereas Cascadura Ikeda is a traditional sweet bell pepper bred in Brazil.

### Metabolic analyses of fruits

Flowers were tagged at anthesis and samples of fruit pericarp and placenta were collected at midday after 60 days. The pericarp and placenta from seven fruits per genotype were collected from different plants and used for the analyses. Samples were immediately frozen in LN_2_ and stored at − 80 °C. The material was then freeze-dried (Scanvac, Coolsafe 55-4) and ground to a fine powder. The metabolic profile of each sample was determined following previously described procedures^49–51^, with modifications summarized briefly here. Firstly, 10 mg of sample material was mixed with 1 mL of extraction buffer consisting of methyl-*tert*-butyl-ether and methanol (3:1), with an internal standard consisting of 50 μL corticosterone (1 mg mL^−1^ in methanol), 50 μL 1,2-diheptadecanoyl-*sn*-glycero-3-phosphocholine (1 mg mL^−1^ in chloroform) and 50 μL ribitol (1 mg mL^−1^ in water). Samples were then vortexed and incubated in a shaker (1000 rpm) at 4 °C for 45 min. 650 μL of water:methanol (v/v 3:1) were then added to induce phase separation and the samples were centrifuged at 20,000×*g* for 5 min at room temperature. Aliquots were transferred from both the upper (apolar) and lower phase (polar and semi-polar metabolites) to new tubes and dried in speedvac for subsequent metabolic analyses. The dried aliquots were stored at − 80 °C until derivatization or resuspension prior to GC- or LC–MS analyses.

For the analysis of primary metabolites, dried aliquots from the polar liquid phase were initially derivatized with 60 μL of methoxiamine hydrochloride (30 mg mL^−1^ in pyridine) and shaken at 37 °C for 2 h. Sample extracts were then trimethylsylilated with 120 μL of a mix of methyl-*N*-(trimethylsylil)trifluoroacetoamide (MSTFA) containing standards of fatty acid methyl esters (FAMEs), followed by agitation at 37 °C for 30 min. Samples were spun briefly and a volume of 140 μL from each was transferred to fresh glass vials, and 1 μL injected for gas chromatography-time of flight-mass spectrometry (GC-TOF–MS) as described previously^52^. Chromatograms and mass spectra were evaluated using ChromaTOF 1.0 (Leco, www.leco.com) and TagFinder v.4.0, respectively. Cross-referencing of mass spectra was performed with the Golm Metabolome database^53^.

For the analysis of apolar metabolites, dried aliquots from the upper lipid phase were resuspended in 400 μL of acetonitrile: 2-propanol 7:3 (v/v) and 140 μL were then transferred to glass vials for injection into an ultra-performance liquid chromatography system coupled to a Q Exactive mass spectrometer in positive ionization mode (UPLC-MS), according to established protocols^51^. Both for the primary and lipid metabolic datasets, standardized metabolite intensities (standardized by internal standard and weight) were then batch-corrected (ComBat), normalized by g*log* transformation and autoscaled (mean-centered and divided by the standard deviation of each variable) using MetaboAnalyst v5.0^54^. The results for metabolites are reported following the standards suggested in Fernie et al.^55^.

### Estimation of heterosis

Heterosis for relative metabolite abundance in hybrids was evaluated using the following equations^24,56^:

$${\text{Absolute Mid-Parent Heterosis }}\left( {{\text{AMPH}}} \right) \, = {\text{ F}}_{{1}} - \overline{{\text{P}}} ,$$

$${\text{Absolute best parent heterosis }}\left( {{\text{ABPH}}} \right) \, = {\text{ F}}_{{1}} - {\text{ P}}_{{{\text{max}}}} ,$$

where: F_1_: average value of the F_1_ hybrid; $\overline{{\text{P}}}$: average of both parental genotypes; and P_max_: Best parent average value.

Relative heterosis values were further calculated as:

$${\text{Relative mid-parent heterosis }}\left( {{\text{rMPH}}} \right) \, = \, ({\text{AMPH }}/\overline{{\text{P}}} ) \times {1}00,$$

$${\text{Relative best parent heterosis }}\left( {{\text{rBPH}}} \right) \, = \, \left( {{\text{ABPH }}/{\text{ P}}_{{{\text{max}}}} } \right) \times {1}00.$$

Reciprocal effect for the hybrids was calculated as the difference between the values of the reciprocal hybrids, e.g.: (HAB × JAL) − (JAL × HAB). Absolute heterosis and reciprocal effect values were subjected to ANOVA, followed by Scheffe’s test to verify the significance of each value individually.

### Statement on plant material

The plant material in this manuscript complies with relevant institutional, national and international guidelines and laws and can be obtained from seed companies worldwide.

## Supplementary Information

Supplementary Information.

## Data Availability

All data generated or analysed during this study are included in this published article and its Supplementary Information files.
